# HEK293 cell culture media study: increasing cell density for different bioprocess applications

**DOI:** 10.1186/1753-6561-7-S6-P51

**Published:** 2013-12-04

**Authors:** Leticia Liste-Calleja, Martí Lecina, Jordi Joan Cairó

**Affiliations:** 1 Chemical Engineering Department, Universitat Autònoma de Barcelona, Cerdanyola del Vallès, 08193, Spain

## Background

The increasing demand for biopharmaceuticals produced in mammalian cells has lead industries to enhance bioprocess volumetric productivity through different strategies. Among them, media development is of major interest [1]. According to the increasing constraints regarding the use of animal derived components on industrial bioprocesses but also the drawbacks of its depletion from cell culture [2], the main goal of the present work was to provide different cell culture platforms which are suitable for a wide range of applications depending on the type and the final use of the product obtained.

## Materials and methods

The cell line HEK293SF-3F6 employed in this study was kindly provided by Dr. A.Kamen, NRC-BRI. The basal media tested were CDM4HEK293, SFM4HEK293 and SFMTransFx-293 (Hyclone, Thermo Scientific) supplemented -when indicated- with FBS (Invitrogen) and/or Cell Boost 5 (80 g/L) (Hyclone, Thermo Scientific). Viable cell density and viability were determined by trypan blue exclusion method and manual counting using an haemocytometer. The adenovirus strain HAdV-5(ΔE1/E3) encoding pCMV-GFP was used for infection experiments. All infections were carried out at MOI≈1 TOI≈0.5 × 10^6^cell/mL in 6-well-plate. Harvesting was performed 48 hpi.

Viral titration was performed by Flow cytometry on a FACS Canto (Becton and Dickinson, Bioscience) by adaption of a protocol previously described [3].

## Results

**Figure 1:**
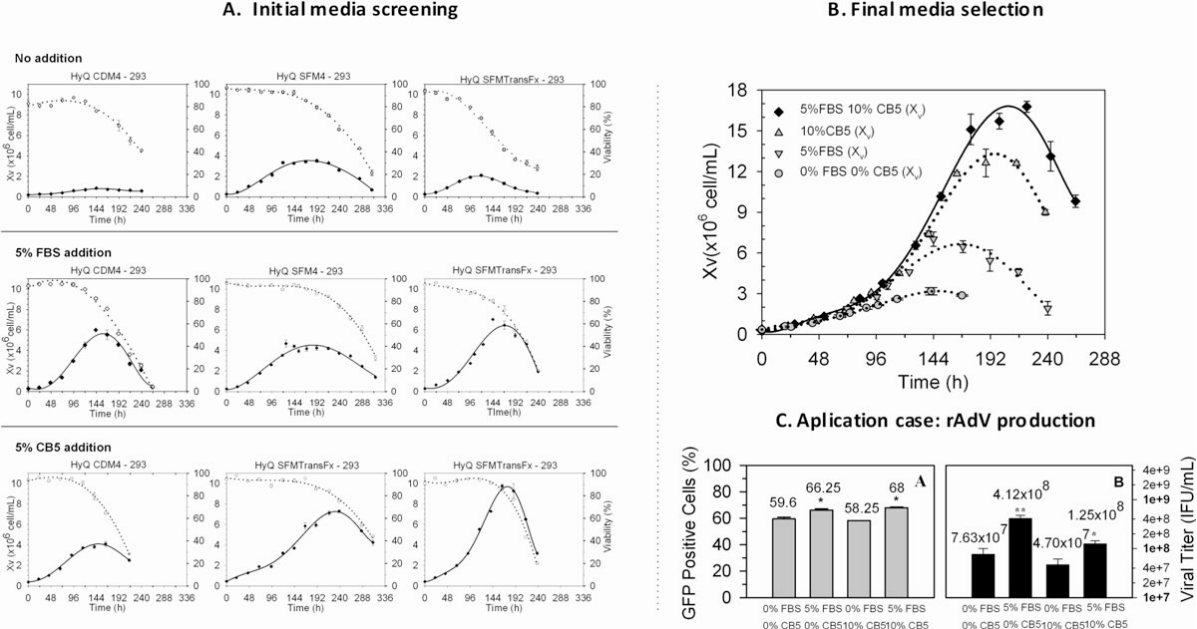
**(A)** Comparison of cell growth profiles of HEK293 cell cultures in serum-free cell media (top panel) and in the same cell media FBS supplemented (middle panel) or CB5 supplemented (bottom panel). **(B)** HyQ SFMTransFx-293 cell cultures with the best concentrations encountered for FBS and CB5 and combination of supplements. **(C) **Evaluation of the effect of supplement addition on efficiency of infection and Viral Titer obtained.

The first part of this work was focused on screening different serum-free cell culture media specifically recommended for HEK293 cell line. As shown in Figure 1A top panel, cultures performed in HyQ SFM4HEK293 and HyQ SFMTransFx-293 showed better cell growth than HyQ CDM4HEK293, reaching maximum cell densities of about 3.5 × 10^6 ^cell/mL, 2 × 10^6 ^cell/mL and less than 1 × 10^6 ^cell/mL respectively. In order to evaluate whether the substitution of critical serum components have satisfactorily been performed in the media tested without affecting cell growth, the addition of fetal bovine serum (FBS) was assessed. FBS depletion was acceptable only in HyQ SFM4HEK293 as the other cell media reached higher cell densities when FBS was added (up to 7-fold increment of Xv_max_). Regarding the screening of Animal derived component free supplements, three chemically defined supplements were tested but only one (Cell Boost 5, onwards CB5) significantly enhanced cell growth. This supplement enabled to reach higher cell densities in all media tested: 2-fold up in HyQ SFM4HEK293 and CDM4HEK293 and 5-fold increment in HyQ SFMTransFx-293 (Figure 1A, bottom panel).

The results obtained so far showed that supplementation of all cell media tested is recommended in order to achieve higher cell density cultures. Among all the conditions, HyQSFMTransFx-293 was the media which supported the highest Xv_max _with both supplements (FBS and CB5). Therefore, this medium was selected for tuning the final concentration of each supplement. Among the studied concentration range for FBS (2.5-10% v/v) and for CB5 (2.5-20%) it was determined that the best conditions were 5% for FBS and 10% for CB5 solution. At these concentrations, Xv_max _achieved were (7.14 ± 0.56*10^6 ^cell/mL) and (12.63 ± 1.76*10^6 ^cell/mL) respectively (Figure 1B). Interestingly, CB5 enabled to extend μ_max _phase while FBS increased μ_max _value, as previously detected in the initial media screening (Table 1). The combination of supplements (5% FBS and 10%CB5) resulted in an Xv_max _as high as 16.77 ± 0.70 × 10^6^cell/mL in batch culture, with an increment in specific growth rate of 15% in comparison to those cultures in which FBS was deprived. Specific growth rate was maintained for 144 h of cell culture.

**Table 1:** Kinetic parameters for HEK293 cell cultures corresponding to the profiles depicted in Figure 1.

		HyQ CDM4HEK293	HyQ SFM4HEK293	HyQ SFMTransFx-293
No adition	Xv_max _(×10^6 ^cell·mL^-1^)	0.85 ± 0.0	3.53 ± 0.21	2.1 ± 0.12
	μ_max _(×10^-2 ^h^-1^)	1.06 ± 0.01	2.46 ± 0.14	2.43 ± 0.03
	t_μ _(h)	96	74	74

5% FBS	Xv_max _(×10^6 ^cell·mL^-1^)	6 ± 0.0	4.67 ± 0.48	7.02 ± 0.06
	μ_max _(h^-1^)	2.61 ± 0.04	2.8 ± 0.05	2.67 ± 0.01
	t_μ _(h)	95	71	72

5%CB5	Xv_max _(×10^6 ^cell·mL^-1^)	4.11 ± 0.33	7.29 ± 0.18	9.75 ± 0.25
	μ_max _(h^-1^)	2.1 ± 0.06	2.06 ± 0.03	2.17 ± 0.03
	t_μ _(h)	92	69	116

From the range of applications in which HEK293 can be used, the work carried out in this work was directed to recombinant adenovirus production. Hence, the evaluation of the effect of supplementation in the cell media selected on adenovirus infection efficiency and final titer obtained was evaluated (Figure 1C). Efficiency of infection was around 63% as expected for an effective infection [4] in all conditions. In regards to adenovirus production, FBS increased it up to fivefold, whereas CB5 supplementation did not affect significantly, and the addition of both supplements almost doubled the viral production in comparison to basal medium. It is proposed that an increment of osmolarity due to the addition of both supplements might explain the slight reduction on productivity in comparison to the addition of FBS solely [5].

## Conclusions

Two culture platforms are proposed for two possible scenarios in basis of the Xv_max _reached: (1) HyQSFMTransFx-293 CB5 supplemented -10% v/v- for animal derived component Free required bioprocesses (Xv_max_= 12.6 × 10^6 ^cell/mL) and (2) HyQSFMTransFx-293 FBS and CB5 supplemented -5% and 10% v/v respectively- for animal derived component containing bioprocesses (Xv_max_= 16.7 × 10^6 ^cell/mL). In both cases, μ_max _and t_μ _values were preserved or even improved with respect to basal media and any of the supplements negatively affected the adenovirus production when compared to non-supplemented infections.
